# Rare occurrence of cryptic 5’ splice sites by downstream 3’ splice site/exon boundary mutations in a heavy-ion-induced *egy1-4* allele of *Arabidopsis thaliana*

**DOI:** 10.3389/fpls.2024.1388040

**Published:** 2024-09-10

**Authors:** Alvin Sanjaya, Ryo Nishijima, Yuki Fujii, Makoto Asano, Kotaro Ishii, Yusuke Kazama, Tomoko Abe, Makoto T. Fujiwara

**Affiliations:** ^1^ Department of Bioscience and Biotechnology, Fukui Prefectural University, Eiheiji, Fukui, Japan; ^2^ Graduate School of Science and Technology, Sophia University, Chiyoda, Tokyo, Japan; ^3^ Ion Beam Breeding Group, RIKEN Nishina Center, Wako, Saitama, Japan

**Keywords:** *Arabidopsis thaliana*, *EGY1*, At5g35220, pre-mRNA splicing, cryptic splice site, heavy ion beam

## Abstract

Pre-mRNA splicing is a fundamental process in eukaryotic gene expression, and the mechanism of intron definition, involving the recognition of the canonical GU (5’-splice site) and AG (3’-splice site) dinucleotides by splicing factors, has been postulated for most cases of splicing initiation in plants. Splice site mutations have played crucial roles in unraveling the mechanism of pre-mRNA splicing *in planta*. Typically, splice site mutations abolish splicing events or activate one or more cryptic splice sites surrounding the mutated region. In this report, we investigated the splicing pattern of the *EGY1* gene in an Ar-ion-induced *egy1-4* allele of *Arabidopsis thaliana*. *egy1-4* has an AG-to-AC mutation in the 3′-end of intron 3, along with 4-bp substitutions and a 5-bp deletion in adjacent exon 4. RT-PCR, cDNA cloning, and amplicon sequencing analyses of *EGY1* revealed that while most wild-type *EGY1* mRNAs had a single splicing pattern, *egy1-4* mRNAs had multiple splicing defects. Almost half of *EGY1* transcripts showed ‘intron retention’ at intron 3, while the other half exhibited activation of 3’ cryptic splice sites either upstream or downstream of the original 3’-splice site. Unexpectedly, around 8% of *EGY1* transcripts in *egy1-4* exhibited activation of cryptic 5′-splice sites positioned upstream of the authentic 5’-splice site of intron 3. Whole genome resequencing of *egy1-4* indicated that it has no other known impactful mutations. These results may provide a rare, but real case of activation of cryptic 5’-splice sites by downstream 3’-splice site/exon mutations *in planta*.

## 1 Introduction

In the eukaryotic gene expression system, the introns of precursor messenger RNAs (pre-mRNAs) are removed before translation of the mRNAs into proteins in a process known as RNA splicing. The molecular machinery of RNA splicing is the spliceosome, a large ribonucleoprotein complex consisting of U2- and U12-dependent types that recognize several highly conserved sequences in introns, namely canonically GU at the 5’ end, an adenine (the branchpoint) located tens of bases away from the 3’ end, and AG at the 3’ end (Alberts et al., 2015). The intron 5’-splice site and the intron 3’-splice site are also termed the splice donor site and the splice acceptor site, respectively. On the basis of biochemical analyses of splicing in animals and yeast and conservation of major splicing machinery components between animals, yeast, and plants, it is postulated that plant pre-mRNA splicing involves similar processes to those of animals, although splice sites in plants are generally recognized via intron definition (Brown and Simpson, 1998; Lorković et al., 2000; Reddy, 2007; Koncz et al., 2012). During splicing, cleavage begins at the 5’-splice site and is followed by the formation of an intron lariat between the 5’-splice site of the intron and a branchpoint adenosine. The exons are then ligated as the intron lariat is released.

Splice site mutations, either spontaneous or induced, have played a crucial role in understanding the mechanisms of pre-mRNA splicing in plants as well as in animals (Brown, 1996; Schuler, 2008). Mutations in canonical sequences of introns can cause a variety of splicing impairments, including inefficient splicing, intron retention (unspliced), exon skipping, or cryptic splicing, which may ultimately result in decreased levels of protein products or production of dysfunctional proteins (e.g., McNellis et al., 1994). Occurrence of cryptic splice sites at either 5’- or 3’-splice sites is often observed in splice site mutants. In most cases, the activated cryptic site is located adjacent to the authentic site, supporting the suggestion that the spliceosome scans along the primary transcript to detect potential splice sites. Accordingly, a mutation in an intron 3’ terminal sequence may trigger a cryptic 3’-splice site(s) but not a 5’ counterpart. The detailed analysis of mRNA splice sites not only can provide information that is useful in recombinant gene construction for basic research, medicine, and biotechnology, but can also reveal potential differences or similarities in the splicing apparatus between organisms that have evolved divergently or convergently. Furthermore, it can advance understanding of the mechanism behind enigmatic alternative splicing phenomena (Reddy, 2007; Staiger and Brown, 2013). In *Arabidopsis thaliana*, analyzed mutations of the intron AG 3’-splice site transition into either AA or GG (Brown, 1996; Schuler, 2008). In either case, activation of cryptic 3’-splice sites is mostly observed, while intron retention, inefficient splicing, or exon skipping can also occur. However, to our knowledge, no model has been reported for the activation of cryptic a 5’-splice site by a 3’-splice site and/or a downstream mutation.

**Figure 1:**
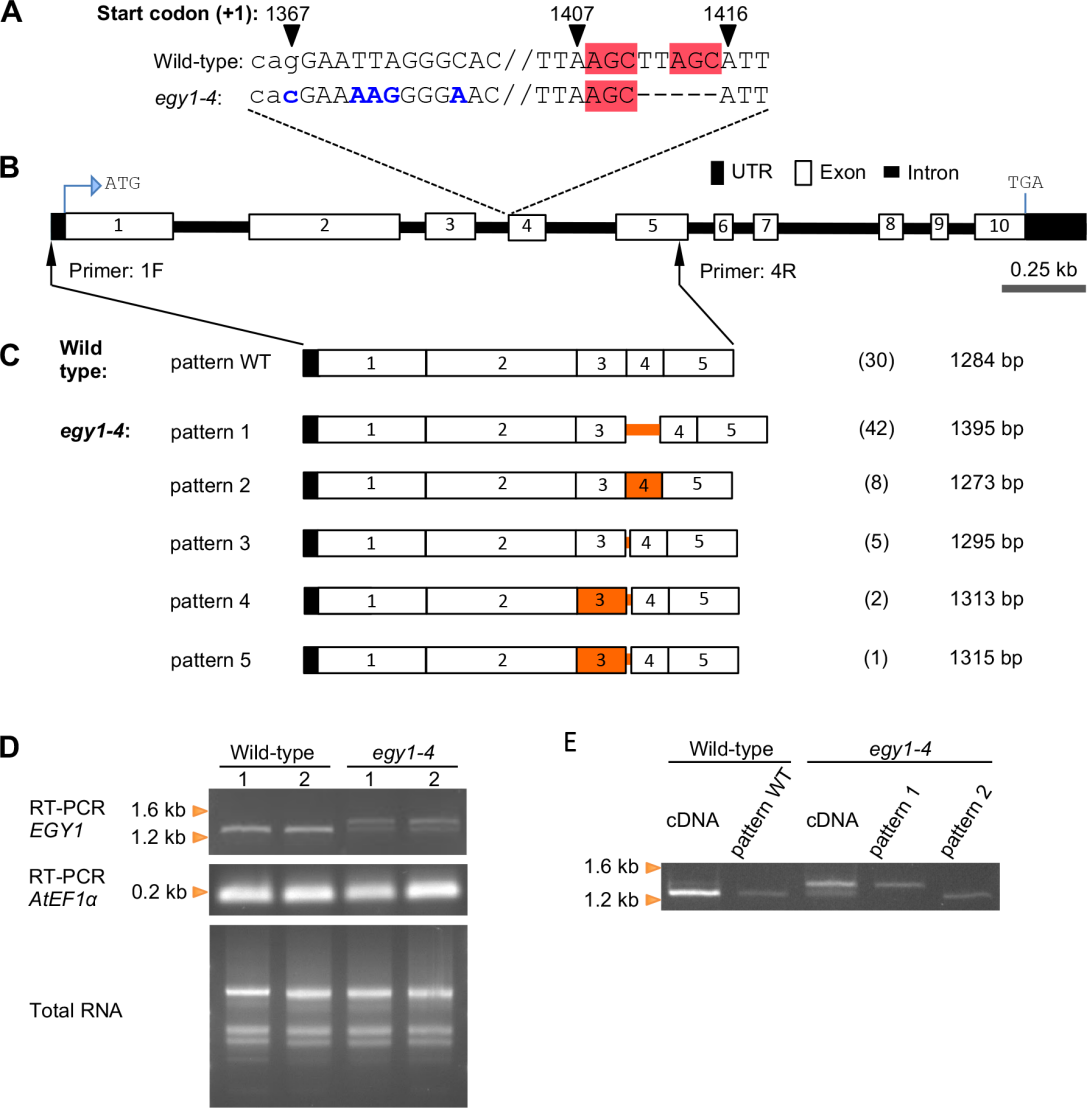
RT-PCR and cDNA cloning analyses of *EGY1* transcripts in the wild-type and *egy1-4* mutant. **(A)** *EGY1* mutation in *egy1-4*. Sequences in blue denote base substitutions, those in the red box denote microhomology, and those in lowercase denote intronic sequences. Numbers on top of certain sequences denote its relative position to the translation start codon (+1). **(B)** Physical map of the *EGY1* gene. ATG, translation start codon; TGA, translation termination codon. Black arrows with primers ‘1F’ and ‘4R’ represent their recognition sites. **(C)** Splicing patterns detected in *egy1-4* versus those in the wild-type. On the right side of each pattern diagram, the count (number in bracket) and the size of the isolated cDNA are shown. Orange lines and boxes indicate the intron and exon regions, showing the different sequences from the wild-type, respectively. **(D)** Electrophoresis of wild-type and *egy1-4* total RNA as well as its corresponding RT-PCR products using primers that bind to *EGY1* or *AtEF1α* (a PCR internal control). For each genotype, two independent leaf materials were used, represented by the numbers ‘1’ and ‘2’. **(E)** Electrophoresis of the PCR products of wild-type and *egy1-4* cDNA together with the PCR products of plasmid clones representing splicing pattern WT, pattern 1, and pattern 2 as templates. For the primer sequence, refer to **Supplementary Table S1**.

In this work, we focused on a mutant allele of *Arabidopsis EGY1* (Ethylene-dependent Gravitropism-deficient and Yellow-green 1, AGI code: AT5G35220), termed *egy1-4*. *EGY1* consists of 10 exons and 9 introns, and encodes a chloroplast-targeted metalloprotease (Chen et al., 2005). The EGY1 protein accumulates in photosynthetic tissues, appears as a single band on Western blots, and possesses proteolytic activity *in vitro*. Loss-of-function mutants of *EGY1* exhibit a pale-green phenotype (Chen et al., 2005; Guo et al., 2008; Li et al., 2012; Yu et al., 2016) and *egy1-4* was no exception. *egy1-4* was generated using heavy-ion beam irradiation, and high-resolution melting analysis and Sanger sequencing revealed that it contains an AG to AC transition at the 3’-end of intron 3, in addition to 4-bp base substitutions and a 5-bp deletion in adjacent downstream exon 4 (Hirano et al., 2012; Sanjaya et al., 2021; see **Figure 1A**). This provided us an opportunity to examine the splicing of *EGY1* mRNAs in *egy1-4*. Here, we report that *egy1-4* exhibits an unusual splicing pattern that has not been previously reported among the defective splicing patterns of *Arabidopsis* generated by intron splice-site mutations (Brown, 1996; Schuler, 2008; The Arabidopsis Information Resource (TAIR) web site [ https://www.arabidopsis.org/]). The preliminary data further underlines the complexity of plant pre-mRNA splicing mechanisms.

## 2 Method

### 2.1 Plant materials and growth condition

*A. thaliana* accession Columbia (Col) served as the wild-type in this study. ^40^Ar^17+^-irradiation-derived *egy1-4* (originally Ar-28-pg1; Hirano et al., 2012) was previously generated in the Col background. The first backcross progeny of *egy1-4* was utilized throughout the study. Seeds were surface sterilized and sown on Jiffy-7 (AS Jiffy Products, Drobak, Norway) or 0.7% (w/v) agar-containing Murashige–Skoog (MS) medium (Wako, Osaka, Japan; Murashige and Skoog, 1962) supplemented with Gamborg’s B5 vitamins and 3% (w/v) sucrose. After vernalization at 4°C for at least 2 days, the sown seeds were placed under white light illumination under a long day conditions (16 h light/8 h dark; approximately 100 µmol m^–2^ s^–1^) at 23°C.

### 2.2 Reverse transcription-PCR and sanger sequencing

Leaves of two-week-old seedlings were harvested for extraction of total RNA using RNeasy Plant Mini Kit (Qiagen, Hilden, Germany) with in-column DNase treatment. Reverse transcription was performed using Invitrogen SuperScript IV Reverse Transcriptase (Thermo Fisher Scientific, Waltham, MA, USA) and an oligo dT(20) primer, followed by PCR using PrimeSTAR GXL DNA polymerase (Takara, Shiga, Japan). Thirty cycles of PCR were run using specific primer sets for *EGY1*, named 1F to 7R (**Supplementary Figure S1** and **Supplementary Table S1**), with each cycle consisting of denaturation at 95°C for 30 sec, annealing at 63°C for 30 sec and elongation at 68°C for 120 sec. The PCR products were then subjected to agarose gel electrophoresis. After confirming the absence of bands resulting from genomic DNA amplification, the bands were extracted from the gel and purified using MinElute Gel Extraction Kit (Qiagen). The purified PCR products were cloned into the pCR-Blunt II-TOPO vector, a component of the Zero Blunt TOPO PCR cloning kit (Thermo Fisher Scientific). Heat-shock–based transformation was performed on DH5α competent cells (Takara). The transformed colonies were selected on LB agar media containing kanamycin (50 μg/mL). Following confirmation by colony PCR, the colonies were further cultured in LB liquid media and plasmids were extracted using a QIAprep Spin Miniprep Kit (Qiagen). The plasmids were mixed with M13 primers and then sent to Eurofins Genomics Co., Ltd. (Tokyo, Japan; https://eurofinsgenomics.jp) for Sanger sequencing.

### 2.3 Amplicon sequencing

Two-step PCR amplicon libraries were constructed. First, the ss-cDNA products were subjected to PCR with *EGY1*-specific primers 8F and 9R (**Supplementary Figure S1** and **Supplementary Table S1**) under the same conditions as described in the previous section. Second, the first PCR products were amplified using the 2nd-F and 2nd-R primers (**Supplementary Table S1**) to incorporate index sequences. The libraries were sequenced using the Illumina NovaSeq 6000 S4 flow cell (Illumina Inc., San Diego, CA, USA; https://www.illumina.com) with other samples at Rhelixa Inc. (Tokyo, Japan). Reads were mapped to the TAIR10 genome assembly using the STAR aligner (version 2.7.10a; Dobin et al., 2013) to detect splicing junctions.

### 2.4 Whole-genome resequencing analysis and determination of mutation loci

Genomic DNA was extracted from leaves using a DNeasy Plant Mini Kit (Qiagen). The extracted DNA underwent sequencing by Macrogen Japan (Kyoto, Japan) using a HiSeq 4000 sequencing system (Illumina) as described previously (Hirano et al., 2015; Kazama et al., 2017). The obtained reads were processed using an automated mutation analysis pipeline (AMAP), as described previously (Ishii et al., 2016). The Arabidopsis TAIR10 release was used as the reference genome. Putative mutations, except those of mitochondria and chloroplast DNA, were further screened for false positives by comparing the mutations in several heavy-ion mutant lines obtained from the same batch of heavy ion beam-irradiated seeds. Mutations that were shared across mutant lines were regarded as false positives and removed from the list of candidates. Next, the candidate mutations were screened further for false positives manually using Integrative Genomics Viewer (IGV) (Robinson et al., 2011).

## 3 Results

**Figure 2:**
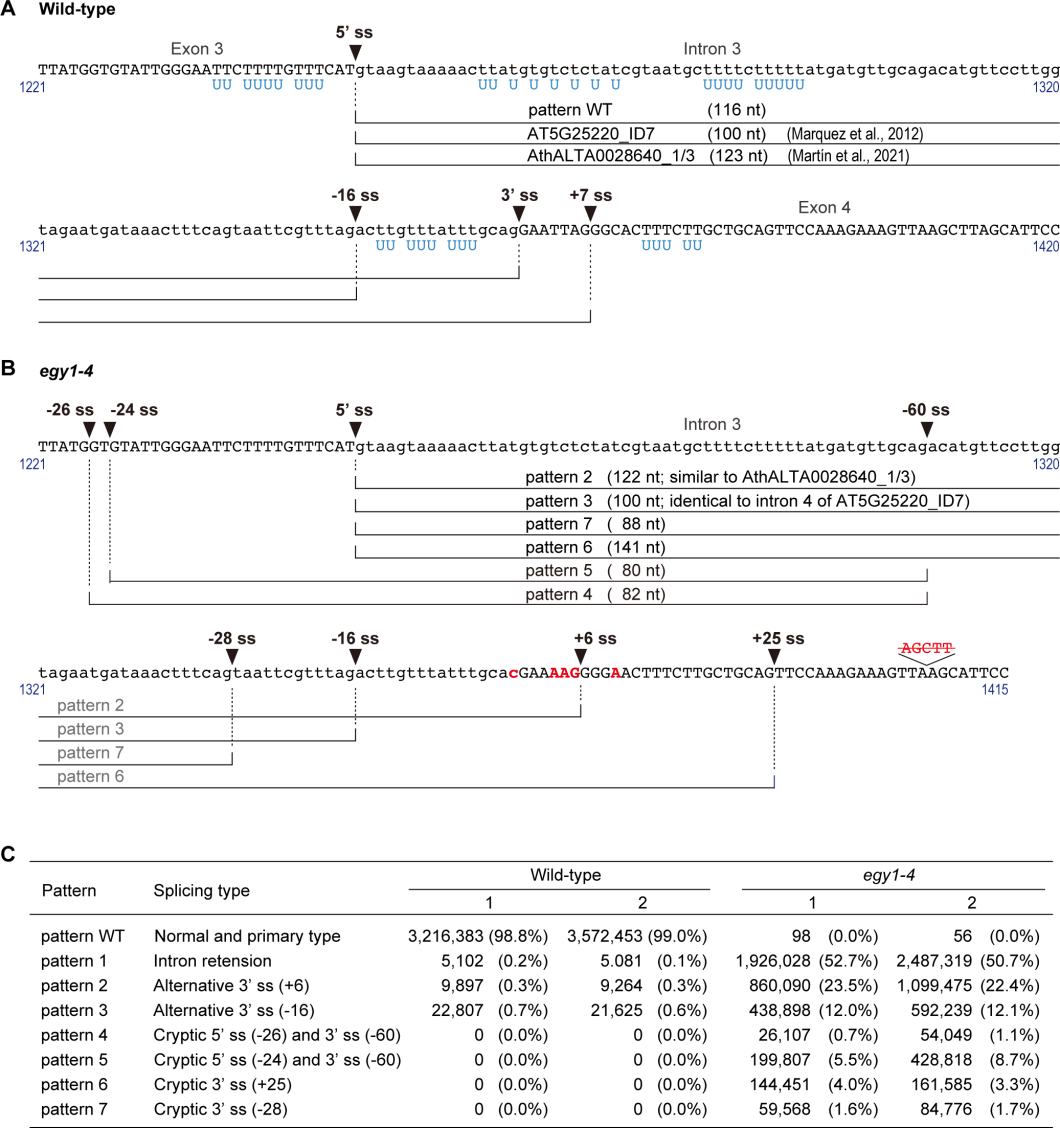
Splice sites in/surrounding intron 3 of *EGY1*. **(A)** Splicing patterns in the wild-type. **(B)** Splicing patterns in *egy1-4*. Sequences in blue denote uracil in U-rich regions of *EGY1* primary transcripts, sequences in red denote base substitutions or deleted nucleotides in *egy1-4*, and those in lowercase denote intronic nucleotide sequences. **(C)** Splicing pattern analysis of *EGY1* intron 3 in wild-type and *egy1-4* by next-generation sequencing. RT-PCR products of *EGY1* from wild-type and *egy1-4* leaf RNAs were analyzed. Read numbers with percentages are shown for wild-type and *egy1-4* samples by two independent experiments. ss, splice site.

First, we checked the splicing profile of *Arabidopsis EGY1* using Araport11 (Cheng et al., 2017) and PastDB (Martín et al., 2021) web sites and compiled the results in **Figure 2A**, **Supplementary Figures S2**-**S5** and **Supplementary Table S2**. *EGY1* mRNAs are preferentially expressed in shoot tissues (Chen et al., 2005), and RNA-seq analyses revealed that *EGY1* has a major mRNA product (At5g35220.1) and at least eight minor splice variants (Marquez et al., 2012; Martín et al., 2021). All the minor splice variants occurred at low frequency (< 3% PSI [percent spliced in]) and the principal mRNA type (At5g35220.1) accounted for >98% of *EGY1* mRNAs over the open reading frame (ORF) region under the normal Arabidopsis growth condition. Therefore, nine introns of *EGY1* were classified into constitutive introns *in silico* (PastDB web site [ https://pastdb.crg.eu/], accessed in Jul 2024). All minor splice variants also contained premature termination codons, which should produce C-terminally truncated EGY1 protein lacking one or more metalloprotease-signature motifs (**Supplementary Figure S3**). Therefore, expression of *EGY1* splice variants represents the ‘regulated unproductive splicing and translation’ process (Filichkin et al., 2010). Together, these results indicate that translation from the principal mRNA species (At5g35220.1) likely yields the main EGY1 product with protease activity in *Arabidopsis*.

To understand whether and how the mutations in the *EGY1* intron 3–exon 4 junction region affect the splicing pattern of *EGY1* in *egy1-4* plants, we investigated a 5’-*EGY1* transcript region encompassing the 5’-UTR and exon 5, in both wild-type and *egy1-4* plants (**Figures 1A, B**). To do this, total RNA was extracted from leaves and subjected to RT-PCR using an oligo dT primer. The resulting ss-cDNA products, which should be enriched from mature (polyadenylated) mRNAs, were subjected to PCR with *EGY1*-specific primers 1F and 4R (**Figure 1B**; **Supplementary Table S1**). Subsequently, cDNA clones were obtained by introducing the PCR products into plasmids and transforming *E. coli* with these plasmids, and their sequences were compared with those in the *Arabidopsis* genome and cDNA databases from TAIR and PlantGDB (Wang and Brendel, 2006). Among 30 cDNA clones derived from the wild-type, only one splicing pattern was observed. All introns in the amplified region (intron 1 to intron 4) contained GU-AG dinucleotides at their borders, and were completely excised (**Figure 1C**). We similarly examined the 3’-region (exon 5 to exon 10) of *EGY1* transcripts using primers 6F and 7R (see **Supplementary Figure S1**), and confirmed that *EGY1* mRNAs in wild-type leaves undergo a constitutive splicing pattern (data not shown). On the other hand, among the 58 cDNA clones of *egy1-4*, we observed five splicing patterns, which used three splice donor sites and three splice acceptor sites (**Figures 1C**, **2B**). The five splicing patterns are described as follows:

*Pattern 1.* This pattern was observed in 42 cDNA clones. Intron 3 was completely unspliced (intron retention type), while other introns were correctly spliced. The size of intron 3 is 116 nt, and a premature termination codon occurs within the retention sequence for the production of a C-terminally truncated EGY1 protein (**Figure 2B**; **Supplementary Figure S3**).

*Pattern 2.* This pattern was found eight times and had the same 5’-splice site as the wild-type, but the 3’-splice site was shifted 6 nt downstream (AG↓GG). This should also result in the production of a C-terminally truncated EGY1 protein, due to a frameshift event at exon 4 (**Figure 2B**; **Supplementary Figure S3**).

*Pattern 3.* This pattern was observed in five cDNA clones and had the same 5’-splice site as the wild-type, but the 3’-splice site was shifted 16 nt upstream (AG↓AC). This should introduce a 16 nt-extension to the 5’-end of exon 4, yielding a frameshift and a premature termination codon downstream, when compared with wild-type exon 4 (**Figure 2B**; **Supplementary Figure S3**).

*Pattern 4.* This pattern was observed in two clones. Both the 5’- and 3’-splice sites were shifted upstream by 26 nt (TG↓GT) and 60 nt (AG↓AC), respectively, from the wild-type splice sites. This should also cause amino acid changes from the 303^rd^ position of EGY1, and translational stop by a premature termination codon (**Figure 2B**; **Supplementary Figure S3**).

*Pattern 5.* This pattern was observed in one clone and had the same 3’-splice site as *Pattern 4* (AG↓AC) but the 5’-splice site was located 2 nt further downstream (GT↓GT). This results in a 24 nt deletion of exon 3 and a 60 nt extension to the mutated exon 4, which should stop translation downstream of the new splice site (**Figure 2B**; **Supplementary Figure S3**).

When amplifying the 3’-region (exon 5 to exon 10) of *EGY1* cDNAs using the same template (ss-cDNAs) and a different primer pair (primer 6F and 7R; see **Supplementary Figure S1**), no aberrant splicing patterns were detected in the region downstream of exon 5 (intron 5 to intron 9) among the 38 cDNA clones derived from *egy1-4* (**Supplementary Figure S1**). In addition, throughout the experiments, we observed no harmful effects of the amplified cDNAs on the growth of *E.coli* colonies.

Agarose electrophoresis of the RT-PCR product of the *EGY1* transcript from *egy1-4* revealed two main bands, whereas it revealed only one main band for the wild-type (**Figure 1D**). The electrophoresis band patterns remained consistently reproducible even when other primer pairs, bounding slightly shifted sites, were used (primers 2F and 5R as well as primers 3F and 5R; **Supplementary Figure S1**; data not shown). Furthermore, PCR analysis using two *egy1-4* cDNA clones from *Pattern 1* and *Pattern 2* indicated that at least *Pattern 1* corresponded to the larger band in the RT-PCR product of *egy1-4* (**Figure 1E**). Based on the band intensities on the agarose gel, we estimated that there was probably a greater than 50% chance that intron 3 is not spliced in the mutant (**Figure 1D**).

To more precisely investigate the splicing patterns of *EGY1* at intron 3, amplicon sequencing was performed. An *EGY1* region across exon 3, intron 3, exon 4, intron 4, and exon 5 (526 bp at the genomic level and 185 bp at the cDNA level in wild-type) was amplified by two-step PCR using the wild-type- and *egy1-4*-derived ss-cDNAs as templates, and the products were subjected to Illumina next-generation sequencing (NGS). On average, 3.86 M reads were obtained for each sample. In the wild-type, the canonical splicing pattern accounted for 98.92% of reads, which was consistent with the PSI values of the leaf samples in the PastDB (**Supplementary Figure S4C**). On the other hand, *Patterns 1*, *2*, and *3* were observed in 0.15%, 0.28%, and 0.65% of reads, respectively. In the *egy1-4* mutant, *Patterns 1*, *2*, *3*, *4*, and *5* accounted for 51.69%, 22.97%, 12.04%, 0.91%, and 7.11% of reads respectively, which was roughly similar to the results of cDNA cloning analysis (**Figures 1C**, **2C**). In addition, two novel splicing patterns were detected in the amplicon analysis of *egy1-4*:

*Pattern 6.* This pattern was found in 3.62% of reads and had the same 5’-splice site as the wild-type, but the 3’-splice site was shifted 25 nt downstream (AG↓TT) (**Figure 2B**). This should result in the loss of the metal-binding HEXXH motif, but conservation of most of the C-terminal sequence including the termination codon of the wild-type (**Supplementary Figure S3**).

*Pattern 7.* This pattern was observed in 1.68% of reads and had the same 5’-splice site as the wild-type, but the 3’-splice site was shifted 28 nt upstream (AG↓TA) (**Figure 2B**). This should introduce a 28 nt-extension to the 5’-end of exon 4, yielding a frameshift and a premature termination codon downstream, which are absent in wild-type exon 4 (**Supplementary Figure S3**).

Overall, mutations in the *egy1-4* allele caused intron retention in about half of *EGY1* transcripts, while they affected 3'-splice sites in the other half. *Patterns 2* to *7* followed the GU-AG rule, and *Patterns 2* and 3 corresponded to two minor splicing variants in the wild-type, whereas *Patterns 4* and *5* exhibited cryptic 3’-splice sites accompanying cryptic 5’-splice sites.

We conducted genome resequencing of *egy1-4* to detect abnormal RNA splicing, which is expected to occur when genes encoding splicing machinery-related proteins and/or their expression are disrupted (Herdt et al., 2017; Tang et al., 2020). Among the mutations detected, single nucleotide substitutions were found in the promoter regions of six Arabidopsis Splicing Related Proteins (ASRP) genes. No mutations were found in the coding region of any of the 395 listed ASRP genes (http://www.plantgdb.org/SRGD/ASRG/ASRP-home.php, accessed in 2021). In addition to ASRP genes, we detected a homozygous mutation in one gene, heterozygous mutations in six genes (**Supplementary Table S3**), as well as single nucleotide substitutions in promoter regions of other genes. There were no mutations detected within a 100-kb range upstream and downstream from the affected *EGY1* locus. Sanger sequencing further confirmed that there were no mutations in *egy1-4* other than those identified by whole-genome resequencing analysis (data not shown). Our recent crossing experiments confirmed that the mutations at the *EGY1* locus are responsible for the pale-green phenotype in *egy1-4* (Sanjaya et al., 2021). Genome sequencing also revealed that chromosomal rearrangement did not occur *in egy1-4*. Therefore, it is highly probable that *EGY1* gene mutations alone are the cause of this novel splicing phenomenon found in the *egy1-4* allele in this study.

## 4 Discussion

In *Arabidopsis*, systematic genomic and transcriptome analyses since the 1990s have revealed that over 20,000 of approx. 28,000 protein-coding genes have introns (e.g., Goodall and Filipowicz, 1989; Lorković et al., 2000; Wang and Brendel, 2006; Reddy, 2007; Filichkin et al., 2010; Marquez et al., 2012). >70% of introns are < 200 nt in length and have poorly conserved sequences. Besides general U-snRNPs and SR proteins, chromatin status, promoter activity, exon-intron structure, and chemical modification of pre-mRNAs are known to participate in the control of eukaryotic splicing in a gene-dependent manner (e.g., Dolata et al., 2015; Pajoro et al., 2017; Jabre et al., 2021). These results highlight the complexity of splicing mechanisms and the importance of research at the single-gene level in plants.

In this report, we investigated the splicing patterns of *EGY1* in an Ar-ion-mutagenized *egy1-4* mutant, to uncover splicing defects. Intron retention was the primary defect (*Pattern 1*). *Pattern 2* was the second most frequent transcript pattern, and along with *Patterns 3*, *6* and *7*, fits with typical spliceosome behavior, which prefers the next closest recognition element if the authentic one is mutated (Brown, 1996; Schuler, 2008), although 3’-splice sites in *Patterns 2* and *3* occurred at very low frequency in the wild-type (**Figure 2C** and **Supplementary Figure S2**). However, *Patterns 4* and *5* were novel since their 5’-splice sites were altered (**Figure 2**); upstream 5’ cryptic sites located 24–26 nt away from the authentic 5’-splice site of intron 3 in the wild-type were used together with an upstream 3’ cryptic site. Although these two splicing patterns appeared plausible from the mechanistic point of view, i.e., the excised 80–82 nt region contained 5’-splice site sequences complimentary to U1 snRNA (Golovkin and Reddy, 1996; McCullough et al., 1996) and had an enriched AU content (~70%), they did not fit any of the defective splicing patterns determined so far in *A. thaliana* (Brown, 1996; Schuler, 2008). Up to 10% of *EGY1* pre-mRNAs underwent these unusual splicing events in *egy1-4* (**Figure 2C**), provided a rare but real case of a constitutive 5’-splice site being altered to accomodate downstream 3’-spice site/boundary mutations.

So far, no previous studies have linked any of the seven genes listed in **Supplementary Table S3** to the normal function of the splicing mechanism. Thus, other than a causal relationship between the *EGY1* mutations in *egy1-4* and the occurrence of splicing *Patterns 4* and *5*, another possibility is that one or a few of the listed genes (**Supplementary Table S3**) have an unknown role in the splicing process, although we think this possibility unlikely.

Among the seven detected *EGY1* splicing patterns in *egy1-4*, *Pattern 1* – the inclusion of intron 3 in the mature transcript – was found to be the most common. The inclusion of intron 3 (116 nt) would annul the normal translation of the downstream conserved sequences, including the GNLR motif, HEXXH motif, and NPDG motif, as well as the sequences of many transmembrane domains (Chen et al., 2005). Similarly, the other patterns in *egy1-4*, *Patterns 2–5* and *7* would also express short EGY1 proteins lacking the C-terminal domain (**Supplementary Figure S3**). An exception is *Pattern 6*, which will only lack a short region at 312–324 aa. Nevertheless, this deleted region contains the HEXXH motif, which is essential for the proteolytic function of metalloproteases (Chen et al., 2005). Therefore, all the EGY1 proteins produced in *egy1-4* should be dysfunctional.

A G to C transversion at the 3’-splice acceptor site has been documented in humans and is known to cause exon skipping (Bespalova et al., 1997); however, despite occasional occurrences in *H. sapiens*, *Drosophila melanogaster*, and *Oryza sativa* and even *A. thaliana* (Schuler, 2008; Wang and Brendel, 2006), this mutation has not been well examined in plants. *egy1-4* did not seem to utilize the mutated 3’-splice site (i.e., the AT–AC combination) for excising intron 3. This could be attributed to the absence of the UCCUU(A/G)A(C/U) branchpoint sequence, one of the compatible consensus sequences used during noncanonical splicing events (Schuler, 2008), in intron 3. On the other hand, only certain splicing donor and acceptor site combinations were retrieved from *egy1-4* transcripts (e.g., the 5’-splice site of *Patterns 4* and *5* never paired with the 3’-splice site of *Patterns 2* and *3*), suggesting regulatory constraints involving optimal distances between available branchpoint sites, AU-rich elements, and splice sites, as well as compatibility of 3’- and 5’-splice sites with their respective spliceosomal RNAs (McCullough et al., 1996). The fact that the size of the excised introns in *egy1* transcripts ranged from 80-140 bp (**Figure 2**), aligning closely with the typical average length of plant intron (Brown, 1996; Reddy, 2007; Schuler, 2008; Marquez et al., 2012), may suggest the conservation of intron length as a key index.

The results of the current study suggest a link between the recognition of 5’- and the recognition of 3’-splice sites in plant introns. Although the processes underlying the splicing patterns in *egy1-4* are unknown, recent research into pre-mRNA splicing could provide hints. In the chromosomal context, *EGY1* is a multi-exon gene and the *egy1-4* mutations were all located in its middle ORF. A large portion of pre-mRNA splicing events in *Arabidopsis*, including alternative splicing, were recently shown to occur co-transcriptionally, in a manner that correlated with intron number (Li et al., 2020; Zhu et al., 2020). Splicing events at the *EGY1* locus in both wild-type and *egy1-4* plants might also occur during transcription. According to the general splicing model (Brown, 1996; Meyer et al., 2015), the initial stage of splicing is the recognition of the future 5’- and 3’-splice sites by U1 snRNP and U2AF, respectively. U1 snRNP binds to the 5’-splice site via base pairing between U1 snRNA and the 5'-splice site, while U2AF binds to the 3’-splice site and its upstream polypyrimidine tract via direct protein-RNA interactions. In the case of *egy1-4*, mutations in the *EGY1* intron 3/exon 4 boundary region might be recognized prior to the recognition of potential 5’-splice sites by U1 snRNP. Considering the relatively short length of *EGY1* intron 3, the initial failure of U2AF to bind to the mutated 3’-splice site might result in its repositioning or transposition to an appropriate 3’-splice site(s), resulting in the activation of a 3’-cryptic splice site 60-nt upstream of the authentic acceptor site (**Figure 2B**). Upon or after this, the U1 snRNP might recognize upstream potential 5’-splice sites (-24, -26). With regard to the possibility of cooperative recognition and definition of both 5’- and 3’-splice sites, previous studies have reported that an *Arabidopsis* homolog of U2AF^35^, a U2AF subunit that binds to the 3’-splice site and forms a complex with its partner protein U2AF^65^, interacts with the splicing regulator SR45 and a U1 snRNP component U1-70K (Day et al., 2012). The interaction between U2AF^35^ and U1-70K is plant-specific, and it is tempting to speculate that this interaction underlies the cryptic splice site determination in *egy1-4*. Also, recent genetic analyses of *Arabidopsis* revealed important functions of RBP45d (a homolog of human TIA-1), PRP39a (a homolog of human U1 auxiliary protein), and PRP8a (a homolog of yeast U5 snRNP protein) in the organization of the 5’-splice site selection (Kanno et al., 2020; Huang et al., 2022; Llinas et al., 2022). RBP45d binds to U1C, a core component of U1 snRNP, PRP39a, and a U-rich sequence (Huang et al., 2022). Since several U-rich sequences exist in the *EGY1* exon 3-intron 3 region (**Figure 2A**, marked in blue letters), these factors might also have roles in 5’-splice site selection in *EGY1* intron 3.

As a future perspective, one of the highlighted areas in plant splicing was its physiological significance (e.g., Staiger and Brown, 2013; Godoy Herz et al., 2019; Martín et al., 2021). In fact, heat treatment for 6h increased the alternatively spliced *EGY1* transcripts at intron 3 (**Supplementary Figure S5**). While the activation of alternative 5’-splice sites in *EGY1* intron 3 has not been observed in the wild-type, it will be interesting nonetheless to determine whether or how various stresses affect 5’-splice site selection in *egy1-4*. Moreover, future studies will be also needed to examine whether the 3’-splice site mutation is sufficient for the activation of 5’-cryptic splice sites in *EGY1* intron 3 in *egy1-4.*

Finally, the detection of unusual splicing patterns by the *egy1-4* mutation may emphasize the effectiveness of heavy ion beam irradiation in creating novel mutations. This effectiveness of this type of mutagenesis is partly influenced by the linear energy transfer (LET) characteristics of the ions in heavy-ion beams, which determine the nature of the mutations. Specifically, carbon ions, with relatively lower LET, tend to induce smaller mutations, typically disrupting a single gene, similar to that observed after ethyl methane sulfonate-based mutagenesis (Kazama et al., 2011). By contrast, heavy-ion beams with higher LET values, such as those from argon ions, cause large and complex mutations, including chromosomal rearrangements (Kazama et al., 2017). While the comprehensive potential of this mutagenesis technique in generating novel phenotypes is yet to be fully explored, the work in this study exemplifies one way in which this technology can be used to induce specific mutations. This approach could potentially be applied to a wide range of commercially cultivated plants, which would expand the scope of obtaining unexpected and valuable mutants.

## Data Availability

The datasets presented in this study can be found online at: https://www.ddbj.nig.ac.jp, DRR538664 (DDBJ Sequenced Read Archive) or PRJDB17541 (BioProject).
